# Collaborate widely and participate actively

**DOI:** 10.4102/sajid.v37i2.371

**Published:** 2022-02-14

**Authors:** Charles Feldman

**Affiliations:** 1 Department of Internal Medicine, Faculty of Health Sciences, University of the Witwatersrand, Johannesburg, South Africa

My current designation, since January 2018, is the Distinguished Professor of Pulmonology, Department of Internal Medicine, Faculty of Health Sciences, University of the Witwatersrand (Wits), Johannesburg, South Africa (SA). Prior to that, I was Professor of Pulmonology and Chief Physician, Department of Internal Medicine, Charlotte Maxeke Johannesburg Academic Hospital and Wits for > 20 years. My entire career has been spent in academic medicine, and my most important pointers to anyone wishing to have a successful career in academia as a clinician-scientist, based on my own experiences, are indicated in the following section.

## Find yourself a suitable mentor to guide you along your career path

Having completed my undergraduate degree, registrar time in Internal Medicine and the Fellowship of the College of Physicians of South Africa (FCP[SA]), I approached my first mentor, Professor Thomas Bothwell, the Academic Head of Medicine, in those days. I asked him to advise me on what next steps I should follow to develop an academic career.

## Complete a Doctor of Philosophy, so you would be able to supervise others who wish to complete theirs

Professor Bothwell advised me to complete my Doctor of Philosophy (PhD), which I did successfully in 1991, with a thesis on the topic of community-acquired pneumonia (CAP).

## Spend time doing research in a successful research unit, preferably in an International Centre

Professor Bothwell’s next recommendation was for me to do postgraduate research in an overseas institution, which I undertook, as a Research Fellow and Honorary Senior Visiting Colleague (Host Defence Unit, Department of Thoracic Medicine, National Heart and Lung Institute, and Royal Brompton Hospital, London, United Kingdom [UK]). I strongly believed that this experience in basic science research allowed me to become a successful clinician-scientist. I learnt many laboratory-based skills, which I was able to utilise back in SA for my research over many years.

## Participate actively in local and international societies and their activities and congresses

I have been active for many years in both national and international scientific societies. These include the South African Thoracic Society (President of the Society between 1996–1997 and 2001–2003) and more recently the Federation of Infectious Diseases Societies of Southern Africa (FIDSSA; serving on the Council as the Editor-in-Chief of the *Southern African Journal of Infectious Diseases* [SAJID]). I was elected as an Honorary Fellow of the South African Thoracic Society in 2009. Internationally, I was elected as a Foundation Fellow of the European Respiratory Society in 2014 and a Fellow of the American Thoracic Society in 2018 and has been active particularly in the latter society, in which I have held leadership positions.

## Collaborate widely, both within your discipline, across disciplines and with international researchers

The experience in the UK also taught me the immense power of collaborative research, which unfortunately was relatively uncommon in SA at that time but is now the norm. On my return from the UK, I teamed up with Professor Ronald Anderson, the lead scientist in the Department of Immunology, University of Pretoria, and his team. This led to a career-long and incredibly productive partnership across universities. While continuing my clinical studies in CAP during this time, I also personally undertook laboratory-based research in Professor Anderson’s laboratory. Much of this work went towards my DSc degree (senior doctorate), which I was awarded in 2009.

One aspect of academia that is proving increasingly more challenging is securing research funding. Here, once again, the importance of collaboration comes to the fore, with many funding sources, such as the National Institute of Health (NIH), increasingly structuring requests for proposals to favour interdisciplinary research teams.

## Journey into infectious diseases

One aspect of my narrative that will be different from those of others is that my specialist training was as a pulmonologist and/or intensivist, not infectious diseases (ID), yet I wandered into the ID discipline. My interest in respiratory infections, particularly pneumococcal infections, was piqued early in my career by having to manage relatively young, previously healthy patients who developed pneumococcal pneumonia during winter. Many were admitted to the intensive care unit (ICU) at Hillbrow Hospital, where I trained, and once they required mechanical ventilation, they had a 50% mortality. My time in the UK allowed me to do basic research on the pneumococcus using microbiological and other laboratory techniques. Soon after my return to SA, I moved across to the Charlotte Maxeke Johannesburg Academic Hospital as the Academic Head of Pulmonology at Wits. There, in the early 2000s, ID became one of the last disciplines to attain sub-specialty status. I was asked to assist in running the newly established IDs Ward, and in training of the initial Fellows, since there were few other trained ID specialists at that time to do so. In 2011, I was humbled to be awarded Honorary Life Membership of FIDSSA, for my national and international contributions to IDs, and to FIDSSA, mainly as Editor-in-Chief of SAJID.

